# PHIDA: A High Throughput Turbidimetric Data Analytic Tool to Compare Host Range Profiles of Bacteriophages Isolated Using Different Enrichment Methods

**DOI:** 10.3390/v13112120

**Published:** 2021-10-21

**Authors:** Carlos E. Martinez-Soto, Stevan Cucić, Janet T. Lin, Sarah Kirst, El Sayed Mahmoud, Cezar M. Khursigara, Hany Anany

**Affiliations:** 1 Guelph Research and Development Centre, Agriculture and Agri-Food Canada, Guelph, ON N1G 5C9, Canada; cmarti37@uoguelph.ca (C.E.M.-S.); scucic@uoguelph.ca (S.C.); janet.lin@agr.gc.ca (J.T.L.); kirsts@uoguelph.ca (S.K.); ckhursig@uoguelph.ca (C.M.K.); 2 Department of Molecular and Cellular Biology, College of Biological Science, University of Guelph, Guelph, ON N1G 2W1, Canada; 3 Faculty of Applied Science and Technology, The Sheridan College Institute of Technology and Advanced Learning, Oakville, ON L6H 2L1, Canada; elsayed.mahmoud@sheridancollege.ca

**Keywords:** bacteriophage, isolation protocol, host range, phage biocontrol, phage therapy, phage enrichment, lytic phages

## Abstract

Bacteriophages are viruses that infect bacteria and are present in niches where bacteria thrive. In recent years, the suggested application areas of lytic bacteriophage have been expanded to include therapy, biocontrol, detection, sanitation, and remediation. However, phage application is constrained by the phage’s host range—the range of bacterial hosts sensitive to the phage and the degree of infection. Even though phage isolation and enrichment techniques are straightforward protocols, the correlation between the enrichment technique and host range profile has not been evaluated. Agar-based methods such as spotting assay and efficiency of plaquing (EOP) are the most used methods to determine the phage host range. These methods, aside from being labor intensive, can lead to subjective and incomplete results as they rely on qualitative observations of the lysis/plaques, do not reflect the lytic activity in liquid culture, and can overestimate the host range. In this study, phages against three bacterial genera were isolated using three different enrichment methods. Host range profiles of the isolated phages were quantitatively determined using a high throughput turbidimetric protocol and the data were analyzed with an accessible analytic tool “PHIDA”. Using this tool, the host ranges of 9 *Listeria,* 14 *Salmonella*, and 20 *Pseudomonas* phages isolated with different enrichment methods were quantitatively compared. A high variability in the host range index (*HR_i_)* ranging from 0.86–0.63, 0.07–0.24, and 0.00–0.67 for *Listeria, Salmonella,* and *Pseudomonas* phages, respectively, was observed. Overall, no direct correlation was found between the phage host range breadth and the enrichment method in any of the three target bacterial genera. The high throughput method and analytics tool developed in this study can be easily adapted to any phage study and can provide a consensus for phage host range determination.

## 1. Introduction

Bacteriophages (phages) are viruses that specifically infect bacteria [1]. With an approximate number of 10^31^ particles, phages are thought to be the most abundant biological entities on Earth [2]. Due to their ability to lyse their bacterial hosts, lytic phages have been applied in a wide variety of scenarios to combat bacterial pathogens, including therapy (as therapeutic and prophylactic) [3,4], biocontrol, sanitation, preservation [5], detection [6], and remediation [7].

The host range of a phage is usually understood as the breadth of bacteria (strains, species or genera) that it can infect [8]. Although this is a straightforward concept, different techniques for determining the host range can produce divergent results, leading to the suggestion that host range can be defined according to the technique used to assess it [8]. Compared to conventional antimicrobials, phages are specific in their bactericidal activity and capable of infecting hosts generally at a strain, species, or in some cases genus level, leaving non-targeted bacteria unharmed [5,9]. This specificity is driven by different factors such as phage-encoded receptor binding proteins, phage–host interactions during infection, phage-resistance mechanisms, and the presence of related prophages or plasmids [8,10,11,12]. Generally, the selection of a phage with broad or narrow host range during isolation depends on the application. For example, broad host range phages are more desirable for therapeutic and biocontrol applications since multiple strains, serovars, or isolates of the target pathogen(s) are often involved in infections or food contamination [13,14,15,16]. Conversely, broad host phages, especially those capable of infecting multiple genera, could affect beneficial microbiota during therapeutic and biocontrol applications [15]. On the other hand, narrow host range and highly specific phages that bind and infect certain bacterial species are useful for the development of specific and sensitive biosensors for pathogenic or indicator bacteria in clinical, veterinary, and food scenarios [17,18,19]. Additionally, a narrow host range phage would also be more suitable for therapeutic applications when targeting a specific clinical bacterial strain [20].

Phage isolation methods are fairly simple and are traditionally performed using one-step enrichment of environmental samples with single or multiple bacterial targets grown under planktonic conditions before isolating individual plaques. These protocols can yield phages with both narrow and broad host range profiles [8,21,22]. More recently, the use of a sequential series of bacterial strains has been proposed as an alternative phage enrichment strategy to boost the likelihood of isolating broad host range phage strains [23]. Although these approaches are useful for phage isolation, no relationship between the enrichment method and the phage host range has been reported. Thus, the process of isolating phage strains with a desired host range remains challenging, requiring intensive labor and an element of chance.

Additionally, there are different reported protocols to determine phage host range, such as spot testing, the efficiency of plaquing (EOP), and liquid culture-based assay [22,24,25]. Among these, spot testing is one of the most reported host range determination techniques [16,25]. It is easy to perform as it requires only a small volume of phage suspension to be spotted on a bacterial lawn, and produces a straightforward binary result of lysis versus no lysis [8]. Although simple, differences in the clarity of the lytic zones can lead to subjective results regarding the killing efficacy of the phage. Furthermore, this method has been shown to overestimate the host range when compared to other methods, such as EOP [25]. Additionally, since this method relies on applying high phage titer, it does not distinguish between productive infection (phage replication) and lysis due to other mechanisms such as abortive infection and lysis from without [26]. On the other hand, the EOP method consists of spotting serially-diluted phage suspensions onto a bacterial lawn to measure the titer of phage progeny of a particular phage infecting a bacterial strain. Then, the EOP is calculated based on the titer observed in the most susceptible strain (EOP = 1) [25]. This method is considered the ‘gold standard’ approach for phage host range determination, despite being time consuming and labor intensive. Furthermore, it does not reflect the lytic activity of a phage in liquid culture. For example, phages showing high EOP may not exhibit similarly strong lytic activity against bacteria grown under planktonic conditions (e.g., slowly adsorbing phages) [25,27,28,29]. Comparatively, liquid culture-based host range assay is a real-time measurement of culture optical density (OD) in the presence of phage at a given multiplicity of infection (MOI) and it is useful to determine the phage virulence, host range, and development of phage resistance [26]. This assay provides information on the bacterial growth kinetics in the presence of phage and allows to associate quantitative values to the virulence and host range of phages [30].

**Figure 1:**
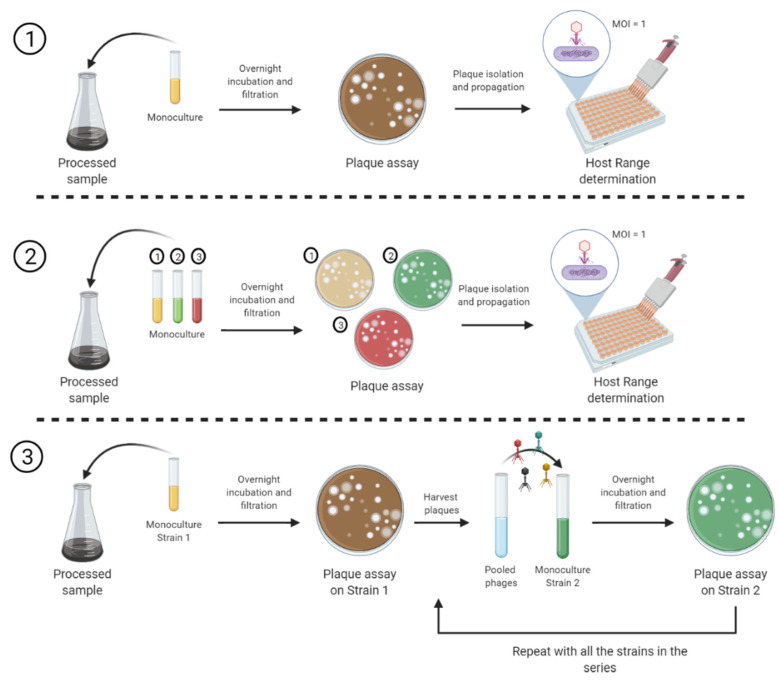
Methods for bacteriophage enrichment and isolation evaluated in this study. (**1**) Single strain (SS): processed sample is enriched with an overnight monoculture and incubated overnight. Mixture is then centrifuged, filtered, and presence of phage is verified by plaque assay on the same host used for enrichment. Plaques are purified and propagated for host range determination using high throughput turbidimetric assay. (**2**) Cocktail enrichment (CE): processed sample is enriched with equal ratios of overnight monocultures (cocktail) and incubated overnight. The mixture is then centrifuged, filtered, and presence of phage is verified by plaque assay on every host used for the enrichment. Plaques from individual hosts are purified and propagated for host range determination using high throughput turbidimetric assay. (**3**) Cyclic sequential (CS) was performed as previously described [31] with some modifications; processed sample is enriched with an overnight monoculture of the first host in the series and incubated overnight. Mixture is then centrifuged, filtered, and presence of phage is verified by plaque assay on the first host. All plaques are pooled and for propagation in the second host of the series. Presence of phage is verified by plaque assay on the second host. The same process is repeated with all the hosts in the series. Plaques recovered from last enrichment are purified and propagated for host range determination using high throughput turbidimetric assay. Created with BioRender.com.

In this study, we compared three different enrichment methods; (1) single strain (SS), (2) cocktail of strains (CE), and (3) cyclic sequential (CS) (Figure 1) to isolate phages against three genera: (1) *Listeria* spp., (2) *Salmonella* spp., and (3) *Pseudomonas* spp., and evaluated the host range profiles of the isolated phages using a high throughput turbidimetric approach that allows for quantitative screening of the effect of phage on the growth of multiple bacterial strains. Additionally, the host range data was analyzed using a rapid and sensitive data analytics tool, PHIDA (http://mahmouel.dev.fast.sheridanc.on.ca/Index_files/downloads.html), and a numerical value (host range index) was assigned to each individual phage to compare the enrichment methods. This method is highly scalable and can be adapted to accommodate multiple phages and bacterial hosts in a single experiment.

## 2. Materials and Methods

### 2.1. Bacterial Strains and Growth Conditions

Bacterial strains used for phage isolation and host range determination are listed in Table S1. All strains were stored at −80 °C in 25% (*v/v*) glycerol. *Salmonella* and *Pseudomonas* strains were streaked out on tryptic soy agar (TSB, Difco^TM^, BD, USA) and incubated overnight at 37 °C. *Salmonella* and environmental *Pseudomonas* strains were grown by inoculating a single colony in tryptic soy broth (TSB, Difco^TM^, BD, USA) and incubated overnight at 37 °C for further use. *Listeria* and clinical *Pseudomonas* strains were cultivated on brain heart infusion agar (BHIA) at 37 °C to obtain single colonies. Individual colonies were inoculated into BHI broth and incubated overnight at 37 °C. Calcium and Magnesium (CM) buffer (0.735 g/L CaCl_2_·2H_2_O: 2.5 g/L MgSO_4_·7H_2_O; 0.05 g/L gelatin; 6 mL/L 1M Tris buffer; pH 7.2 buffer) was used for phage propagation and dilutions.

### 2.2. Sample Enrichment for Phage Isolation

Wastewater was collected from the Waterloo wastewater treatment plant (Waterloo, Ontario, Canada). For phage isolation from wastewater, samples were processed by incubating the primary sludge with 5 mM tetrasodium pyrophosphate (Na_4_P_2_O_7_·10H_2_O) for 15 min at room temperature to free viral particles from the sediment, as previously described [32]. The sample was then centrifuged at 5000× *g* at 4 °C for 20 min and filtered through a 0.22-μm sterile syringe filter (Thermo Fisher Scientific, Mississauga, Canada), the filtrate was stored at 4 °C for further enrichment. The last two steps were omitted for samples collected from food processing facilities. For isolation of *Listeria* phages, run-off water samples from food processing facilities were mixed with 4× concentrated *Listeria* Special Broth (LSB, Bio-Rad^®^, Hercules, CA, USA) to a final concentration of 1× to inhibit the growth of competitor bacteria present in the sample and incubated at 30 °C with moderate agitation for 24 h. Listex P100^TM^ was provided to us by Micreos and was included in this work as a reference phage.

All samples were enriched independently by three protocols as described in Figure 1. Briefly, for (1) Single strain (SS): processed sample was enriched with an overnight monoculture and incubated overnight. The mixture was then centrifuged, filtered through a 0.22-μm sterile syringe filter, and the presence of phage was verified by plaque assay on the same host used for enrichment. (2) Cocktail enrichment (CE): processed sample was enriched with equal ratios of overnight monocultures (cocktail) and incubated overnight. The mixture was then centrifuged, filtered through a 0.22-μm sterile syringe filter, and the presence of phage was verified by plaque assay on every host used for the enrichment. (3) Cyclic sequential (CS) was performed as previously described (Yu et al. 2016) with some modifications. Briefly, the processed sample was enriched with an overnight monoculture of the first host in the series and incubated overnight. The mixture was then centrifuged, filtered through a 0.22-μm sterile syringe filter, and the presence of phage was verified by plaque assay on the first host. Clear plaques were harvested using a sterile micropipette tip and resuspended in 300 μL of CM buffer. Harvested phages were propagated using the soft agar overlay method, as previously described [33]. Presence of phage was verified by plaque assay on the second host. The same process is repeated with all the hosts in the series. The sequence of strains is shown in Table S2. After every enrichment method, clear plaques usually indicative of lytic phages—were harvested, purified, and propagated for host range determination [34,35]. The enrichment methods used for isolating the phages presented in this study are summarized in Table S2.

### 2.3. Host Range Pattern Determination

#### 2.3.1. High Throughput Host Range Characterization in Liquid Culture

**Figure 2:**
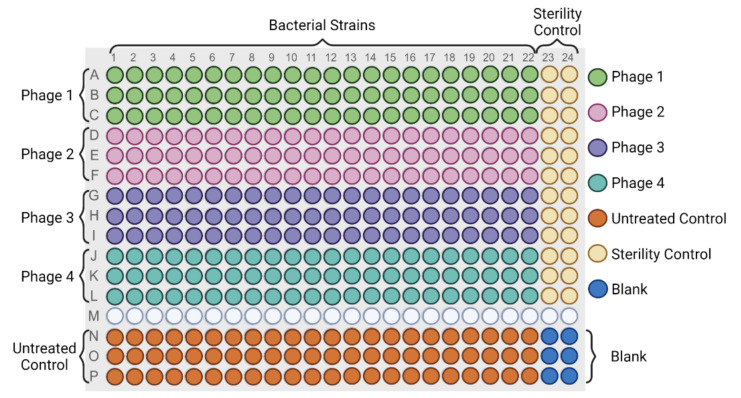
The 384-well plate layout for the high throughput turbidimetric host range determination assay. Columns indicate the number of bacterial strains, and rows, the treatments. Phages 1–4 indicate different phages tested in technical triplicates (50 µL of bacteria dilution + 50 µL of phage dilution). Untreated control (50 µL of bacteria dilution + 50 µL of CM buffer). Sterility control (50 µL of 2 x media + 50 µL of phage dilution). Blank (50 µL of 2 x media + 50 µL of CM buffer). Created with BioRender.com.

Host range of the isolated phages was determined using a high throughput turbidimetric assay in a 384-well plate (Corning, Corning, NY, USA) format (Figure 2). This method allows to test up to four phages against 22 bacterial strains with technical triplicates in a single assay. Phages were titrated and diluted to a final concentration of 7 log_10_ PFU/mL in CM phage buffer. Overnight cultures of all strains (Table S1) were diluted in 2 X TSB for *Salmonella* and environmental *Pseudomonas* isolates and 2 X BHI for *Listeria* and *Pseudomonas* clinical isolates to a final concentration of 7 log_10_ CFU/mL. Bacteria were mixed with phage in a total volume of 100 µL (50 µL of bacteria dilution + 50 µL of phage dilution) to a final theoretical Multiplicity Of Infection (MOI) of 1 (7 log_10_ PFU/mL /7 log_10_ CFU/mL). The plate was incubated at 25 °C for 24 h for *Salmonella* and *Pseudomonas* and 40 h for *Listeria* with continuous double orbital slow agitation using a BioTek Synergy H1 Hybrid Multi-Mode Reader (Winooski, VT, USA). Readings at 600-nm wavelength were taken at 30-min intervals.

#### 2.3.2. PHIDA: Data Analytics Tool for Efficient Host Range Determination

**Figure 3:**
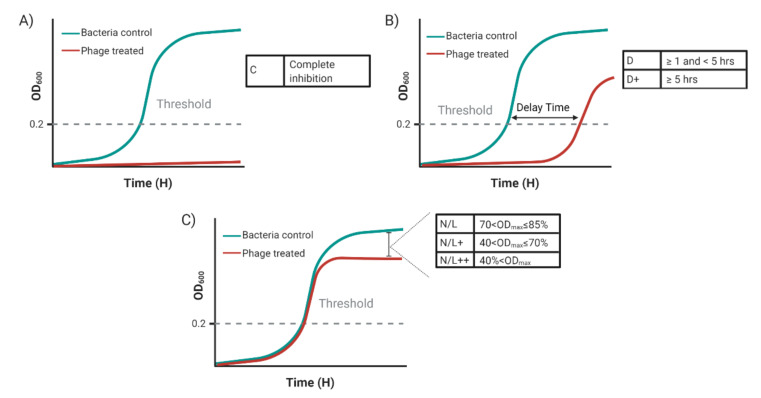
Growth inhibition designations for host range characterization. (**A**) Complete inhibition of the bacterial growth; **C**: sample never reaches detection threshold for the duration of the experiment. (**B**) More than 5 h delay in bacterial growth compared to control; **D+**: time difference to reach detection threshold between sample and control is ≥5 h and <“experiment duration time–detection time of the control”. Less than 5 h delay in bacterial growth compared to control **D**: time difference to reach detection threshold between sample and control is ≥1 and <5 h). (**C**) Time difference to reach detection threshold between sample and control is <1 h. Small effect on bacterial growth endpoint; **N/L**: OD_max_ is 70–85% of control. Moderate effect on bacterial growth endpoint; **N/L+**: OD_max_ is 40–70% of control. Large effect on bacterial growth endpoint **N/L++**: OD_max_ is ≤40% of control. Created with BioRender.com.

Growth curves were analyzed through calculating the time difference between phage treated bacteria cells and untreated control to reach a detection threshold (OD_600_ = 0.2) that represents an early stage of the exponential growth phase of target bacterial strains. Host range was reported according to the growth inhibition efficiency of the phage against a specific host: C = complete growth inhibition, D+ = delay of ≥5 h to reach threshold, and D = delay of growth of <5 h, and N/L, N/L+, or N/L++ according to the endpoint OD_600_ value (Figure 3). The manual detection of these patterns in the data collected from the experiment is time consuming and less accurate. A simple data analytics tool—Phage–host Interaction Data Analyzer (PHIDA)—has been developed to automate the process. The tool facilitates visualizing and cleaning the data, automates identifying replicates that meet a pre-determined OD growth trend, detects the host-range pattern of each replicate automatically, and creates a final report that shows the patterns detected, the average OD, the maximum OD, and the detection time of each sample. The tool was developed using Microsoft Excel because of its ease of use and accessibility features in addition to its powerful analysis/visualization built-in functions. The tool allows researchers to set a group of parameter values for meeting the requirements of various host range experiments. The compatibility of the spread sheets with most analytics software platforms facilitates further analysis and building better host range models through the integration with other software packages.

#### 2.3.3. Implementation of the Data Analytics Tool

The data analytics tool, PHIDA, was implemented using combinations of statistical, lookup, and reference functions of Microsoft Excel in two spreadsheets to determine the effect of phage treatment on bacterial growth in liquid culture. The first sheet is titled data analyzer and outlier detector. This sheet allocates the first nine rows for entering the experiment information, including the experiment parameter values such as the starting time of the experiment, the minimum growth rate, and the interval time in minutes between consecutive readings. The following seven rows are allocated to formulas that calculate the average growth rate of the last three growth rate readings per each sample in the experiment, identify the detection time, detect the growth trend, and classify each sample into a specific designation. The built-in Excel functions that have been used for completing these calculation and analytics tasks include Slope, Indirect, Address, Index, Match, Column, and If. The remaining rows contain the growth rate readings of all samples. The second sheet generates a report based on the data and parameters entered into the first sheet. PHIDA can be easily adapted for other phage–host interaction experiments by reconfiguring the parameter values as needed. The PHIDA tool and a short user’s guide are available at http://mahmouel.dev.fast.sheridanc.on.ca/Index_files/downloads.html. Researchers can download and use the tool for their experiments.

#### 2.3.4. Definition of the Host Range Index

A set of host range measures and metrics were defined and formulated based on bacterial growth inhibition times to quantify the host range of any given phage using a new metric named Host Range index and denoted by *HRi*. The first measure is the maximum inhibition time (*T_max_*). *T_max_* is the duration of the experiment (24 h for *Pseudomonas* and *Salmonella* spp., 40 h for *Listeria* spp.) minus the time required to reach the detection threshold (OD_600_ = 0.2) of the untreated control. The second measure is the normalized value of *T_max_* and named maximum inhibition score (*I_max_*). The maximum theoretical host range score (*HR_max_*) is defined as the number of strains tested for the host range (The *HR_max_* for this work is 22). Using these measures, an inhibition score (*I_s_*) of a phage against a specific bacterial strain can be defined as the quotient of dividing the actual inhibition time (*T_actual_*) by *T_max_:*

$$I_{s\;}\left(25\;{}^{\circ}C,\;24\;\mathrm{or}\;40\;h,\;TSB\;\mathrm{or}\;BHI\right)=\frac{T_{actual}}{T_{max}}$$

Thus, the host range index (*HR_i_*) of a phage is defined as the summation of the phage’s inhibition scores against the panel of bacterial strains divided by HR_max_:

$$HR_{i\;}\left(25\;{}^{\circ}C,\;24\;\mathrm{or}\;40\;h,\;TSB\;\mathrm{or}\;BHI\right)=\frac{\sum I_{s}}{HR_{max}}$$

The *HRi* can be used to facilitate the quantitative comparison of the host range of different phages against the same panel of strains treated in the same conditions. Figure S2 shows an example of these calculations.

## 3. Results

### 3.1. Development of The High Throughput Host Range Assay and PHIDA

**Figure 4:**
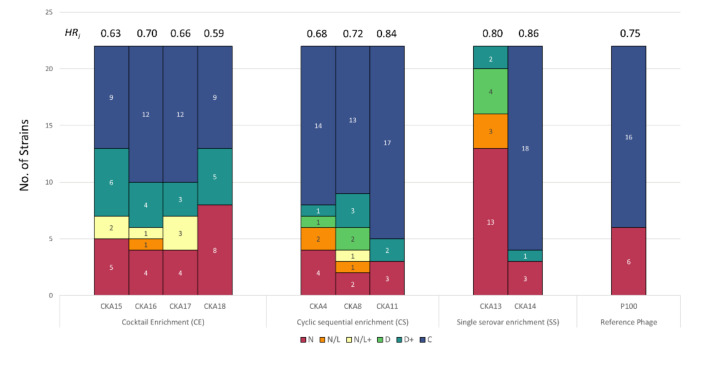
Stacked bar plot showing the host range of *Listeria* phages isolated using different enrichment methods. Designation: C, complete inhibition, D+, >5 h delay to reach exponential phase; D, <5 h delay to reach exponential phase; N, no effect; *HR_i_,* host range index.

**Figure 5:**
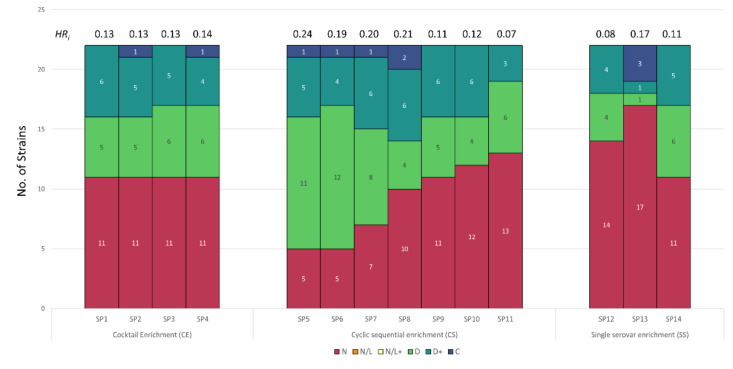
Stacked bar plot showing the host range of *Salmonella* phages isolated using different enrichment methods. Designation: C, complete inhibition, D+, >5 h delay to reach exponential phase; D, <5 h delay to reach exponential phase; N, no effect; *HR_i_,* host range index.

**Figure 6:**
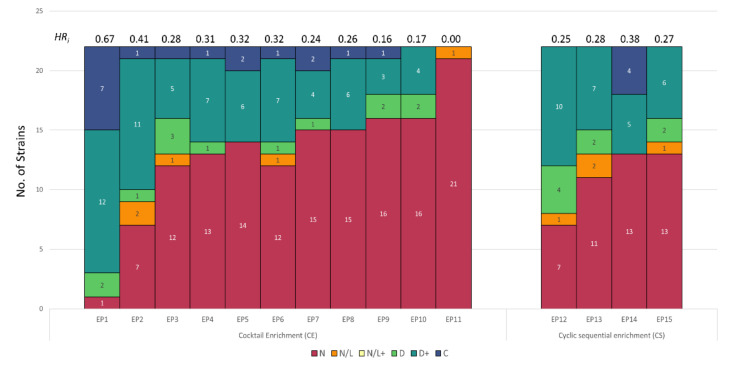
Stacked bar plot showing the host range of phages infecting *Pseudomonas* clinical strains isolated using different enrichment methods. Designation: C, complete inhibition, D+, >5 h delay to reach exponential phase; D, <5 h delay to reach exponential phase; N, no effect; *HR_i_,* host range index.

**Figure 7:**
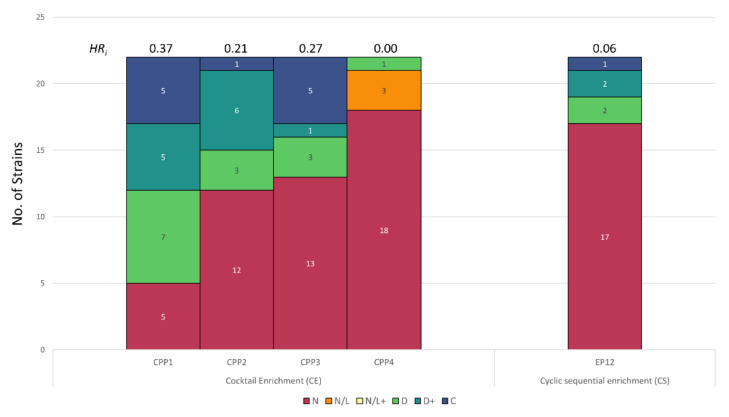
Stacked bar plot showing the host range of phages infecting *Pseudomonas* environmental strains isolated using different enrichment methods. Designation: C, complete inhibition, D+, >5 h delay to reach exponential phase; D, <5 h delay to reach exponential phase; N, no effect; *HR_i_,* host range index.

A high throughput turbidimetric host range determination assay in liquid culture was developed. This assay was performed in a 384-well plate format that allows to test the growth inhibition effect of four different phages against 22 bacterial strains including technical triplicates in a single assay (Figure 2). Bacterial cultures (7 log_10_ CFU/mL) were treated with the phage (7 log_10_ PFU/mL) at a theoretical MOI_input_ of 1. The assay was carried out at 25 °C for 24 h for *Salmonella* and *Pseudomonas,* and 40 h for *Listeria* to achieve consistent optical density results for all tested strains. Using this assay different growth kinetics and inhibition patterns were observed across all tested bacterial strains and phages. The real-time measurements of the growth kinetics provided data to quantitatively designate the growth inhibition efficacy of a phage against a single bacterial host as well as an overall host range for all strains in the test panel (Figure 4, Figure 5, Figure 6 and Figure 7, Tables S3–S6). Moreover, phage–host interactions can be predicted. For instance, in phages with a designation of D or D+, where the bacterial growth is halted for a certain period of time and normal growth is observed thereafter, selection for phage resistant mutants might be the case.

A freely available Microsoft-Excel-based tool called “PHIDA” was developed to rapidly analyze the obtained OD data. The tool is programmed using combinations of statistical, modelling, lookup, and reference functions of Microsoft Excel to determine the effect of phage treatment on bacterial growth in liquid culture. PHIDA allows the user to enter the data, set the experiment parameters, and update the analysis results automatically after each change in the experiment data or the experiment settings. PHIDA analyzes the experiment data to identify outliers for cleaning the data, and automatically identifies replicates that meet a pre-determined OD growth trend that could be set as a parameter. More importantly, PHIDA automatically detects the host–range characterization of each replicate according to the Growth inhibition designations shown in Figure 3. A threshold of detection was designated as OD_600_ = 0.2 as it represents an early stage of the exponential growth in the bacterial strains tested. However, PHIDA allows to set different detection thresholds based on the bacterial strain tested. The tool also automatically generates a report displayed in an additional excel sheet to allow printing it or exporting the report for further analysis by other analysis tools. The correctness of the tool results was demonstrated by the exact match between the tool results and the pre-determined results by lab managers for the same replicates.

### 3.2. Host Range Pattern/Profile of the Isolated Listeria *spp.* Phages

Eighteen *Listeria* phage isolates were obtained from effluent from a meat processing facility and two different poultry plants using the enrichment approaches outlined in Figure 1. A plaque assay was performed by spotting 10 µL of phage suspension of these phage isolates (plus Listex P100™ phage included as a reference) on 19 strains of *Listeria* (Figure S1). Based on the plaque assay host range pattern and degree of lysis on different strains, it was found that at least 9 out of the 18 isolated phages represent unique isolates (Figure S1).

The nine selected phages in addition to the reference Listex P100™ phage were screened using the turbidimetric host range assay, as described above. The analyzed results are summarized in Figure 4 and Table S3. For the phages isolated by the CE method, two (CKA15 and CKA18) completely inhibited 41% of the tested *Listeria* strains and two (CKA16 and CKA17) completely inhibited 55% of them. Between 23–32% of the tested strains were unaffected by these phages and 23–32% were also delayed in growth. Between 59 and 77% of the tested strains were completely inhibited by the presence of phages isolated by the CS method, 9–23% were delayed, and 14–27% were unaffected. For the two phages isolated by single strain enrichment, CKA14 completely inhibited growth in 82% of the strains, delayed growth in 9%, and had no effect on 9%. The other phage isolated by this method (CKA13) delayed the growth of 32% of the tested strains and had no effect on 62% of them. Listex P100™ phage completely inhibited the growth of 73% and had no effect on 27% of the tested strains.

Based on the panel of strains screened, *Listeria* phages that had the highest *HR_i_*-values were CKA14 (SE method) and CKA11 (CS method), with scores of 0.86 and 0.84, respectively. Listex P100™ phage had the third highest *HR_i_* value of the tested *Listeria* phages with a score of 0.75. *HR_i_* values for this set of phages ranged from 0.08 to 0.86 (Figure 4 and Table S3).

### 3.3. Host Range Pattern/Profile of the Isolated Salmonella *spp.* Phages

A total of 14 *Salmonella* phages were enriched and isolated from wastewater using the methods depicted in Figure 1. Host ranges of the isolated *Salmonella* phages are summarized in Figure 5 and Table S4. Three phages with unique host range profiles named SP12, SP13, and SP14 were isolated using the SS method. These phages inhibited the growth of 50%, 23%, and 36% of the tested *Salmonella* serovars (*n* = 22), respectively. SP13 was the only phage that showed complete inhibition against 13% of the tested strains. Interestingly, four phages (SP1, SP2, SP3 and SP4) were isolated using the CE method and considered different phage strains based on their plaque morphology. However, these phages showed almost identical host range profile and delayed the growth of 50% of the same *Salmonella* serovars, suggesting the re-isolation of the same phage. On the other hand, seven phages (SP5, SP6, SP7, SP8, SP9, SP10 and SP11) were isolated using the CS method. SP5 and SP6 phage isolates had similar host range profiles and considered as reisolates of the same phage. The remaining five phages exhibited distinct host range profiles and inhibited the growth of 40–77% of the tested *Salmonella* serovars.

Based on the tested panel of *Salmonella* serovars, the *HR_i_* was calculated for all the isolated phages (Figure 5, Table S4). The *HR_i_* showed to be highly variable among all isolated phages (0.075–0.24) as well as in phages isolated with the same enrichment method ranging from 0.08–0.17 for SS, 0.13–0.14 for CE, and 0.075–0.24 for CS. SP5 and SP8 isolated with CS method exhibited the highest *HR_i_*, 0.24 and 0.21, respectively.

### 3.4. Host Range Pattern/Profile of the Isolated Pseudomonas *spp.* Phages

Two groups of environmental and clinical *Pseudomonas* spp. strains were used for enrichment and isolation of *Pseudomonas* phages from wastewater samples (Figure 6 and Figure 7). Phages infecting environmental isolates of *Pseudomonas* (EP) were isolated using CE and CS enrichment methods as depicted in Figure 1. Due to the complexity and difference in growth conditions between the *Pseudomonas* strains, CE was performed using two different cocktails described in Table S2. Phages infecting clinical isolates of *Pseudomonas aeruginosa* (CP), were isolated using the Liverpool epidemic strain-like (LES-like) isolates [36] (Table S2).

A total of 15 EP phages were isolated and characterized by host range against 21 *Pseudomonas* strains and one *E. coli* strain (Figure 6 and Table S5). Eleven phage isolates (EP1 to EP11) were isolated using the CE method. Phages EP1–3 and EP7–11 had unique host range profiles while EP4, EP5, and EP6 showed very similar host range profiles, suggesting re-isolations of the same phage. Phage EP1 showed a remarkably broad host range by inhibiting the growth of 95% of the tested strains including *E. coli* K-12. Conversely, phage EP11 showed a very narrow host range and did not inhibit the growth of any of the tested strains. Furthermore, four phages (EP12–15) were isolated using the CS enrichment method and had unique host range profiles (36, 50, 40, and 64%, respectively). Interestingly, re-isolations were not observed in the phage isolates isolated with this enrichment method. These phages inhibited the growth of 36 to 64% of the tested strains and EP12 phage had the broadest host range profile.

Contrary to the EP, only five phages were isolated using the CE and CS enrichment methods (Figure 7 and Table S6). Four phages (CP1, CP2, CP3, and CP4) with distinct host range profiles were isolated by the CE method. CE method resulted in the isolation of both broad (CP1, inhibiting 77% of the strains) and narrow (CP4, inhibiting 4% of the strains) host range phages. No re-isolated phages were detected for the clinical *Pseudomonas* isolates compared to the environmental. Interestingly, only one phage (CP5) was isolated using the CS method. This phage was able to infect 18% (4/22) of the strains tested.

*HR_i_* was calculated for EP and CP phages and is summarized in Figure 6 and Figure 7 and Tables S5 and S6. The *HR_i_* of EPs showed great variability ranging from 0.0011 to 0.67 when comparing all enrichment methods. Similar variability was observed in EP isolated with the same enrichment method ranging from 0.11–67.74 for CE, and 25.9–38.89 for CS. Among all EP phages, EP1 isolated with CE method showed the highest *HR_i_* of 67.74. Similarly, the *HR_i_* of CP ranged from 0.80 to 37.84 showing high variation. CP1 isolated with the CE method showed the highest *HR_i_* of 37.84.

## 4. Discussion

Phages are ubiquitous in the world with approximately 10^31^ particles [2]. The specificity of lytic phages to infect and kill bacteria has made them an appealing tool to combat pathogens [3,4,5,6,7]. There are numerous selection criteria for phages suitable for therapy and biocontrol application. Candidate phages should be obligately lytic, lack genes encoding toxins or antibiotic resistance mechanisms, have infection kinetics that favor efficient killing of their hosts, and be capable of killing a wide range of hosts [37]. In particular, the host range criterion is a crucial one to determine the proper application of the isolated phages. For instance, phages with broad spectrum against multiple strains or serotypes are desired for biocontrol applications [16,38,39]. Conversely, in other applications such as detection and diagnosis, phages with a narrow host range are more useful to distinguish between pathogenic and non-pathogenic serotypes or strains of bacteria [19,40].

In the literature, phages are often categorized as narrow and broad host range. These are subjective terms and mainly linked to the number and type of strains tested in every study. For example, Huang et al. [41] reported *Salmonella* phage LPSE1 as a broad host range infecting seven *Salmonella* strains (three different serovars) in a spot test assay. On the other hand, Islam et al. [16] reported *Salmonella* phage LPST4 as a broad host range infecting 38 *Salmonella* strains (13 different serovars) using the same approach, but they also included EOP results. It is reasonable to assume that phage LPST4 has a broader host range compared to LPSE1. However, it is difficult to make a direct comparison between these two phages since different experimental design was used for the host range experiment in both studies. This highlights the importance of having a consistent and quantitative approach to compare and report the host range of a given phage.

Although different enrichment methods for phage isolation have been described, there is still a knowledge gap regarding the relationship between phage host range and the enrichment method applied. Additionally, the lack of a consensus method to determine the host range complicates the comparison between studies [25]. The results obtained from the spot test and EOP can be subjective, especially when considering that different phage traits such as diffusivity, adsorption rate, latent period, burst size, and other factors such as incubation time and media composition can influence the morphology of the phage plaques [42,43,44].

In this study, three different enrichment methods were used for the isolation of lytic phages from various environmental samples against two Gram negative bacterial genera (*Salmonella* and *Pseudomonas*) and one Gram positive genus (*Listeria*). Then, a high throughput screening approach in liquid medium was implemented to determine and compare the host range profiles of the isolated phages with different enrichment methods. This approach can provide a tool for the rapid determination of the phage host range, since it allows to determine the growth kinetics of phage treated bacterial strains. Using the 384-well plate format, the assay can accommodate 22 bacterial strains and four phages in technical triplicates in a single experiment, including the proper controls (Figure 2). Although a straightforward experiment, several technical considerations must be taken when performing this assay. For instance, to avoid bacterial growth before phage application, it is recommended to perform the experiment in a continuous manner (2–3 h using multi-channel pipette) as well as preparing bacterial dilutions right before adding them to the plate. To prevent cross-contamination between wells, a slow agitation speed is recommended during incubation. Alternatively, the volume in the wells can be reduced by 10–20%, which does not affect the results (data not shown). Media evaporation can occur when incubating plates for long periods of time or temperatures above ambient; however, that was not observed in this study (incubation time of up to 40 h, 25 °C). This can vary depending on the equipment, plate, temperature, and incubation time. Should that be the case for other conditions, it is recommended to use sterile membranes to cover the plates (e.g., Breathe-Easy^®^ sealing membranes) to avoid media evaporation. Additionally, temperature plays an important factor in both bacterial metabolism and phage infectivity [45]. For example, the broad host range *Listeria* phages that we have isolated in this study do not produce plaques when incubated at 37 °C. Here, a temperature of 25 °C was used to study the infection of all phages; however, various temperatures can be tested following the same protocol. It is important to highlight that phages selected with this method need to be tested at different temperatures depending on the application, for example, 4–15 °C for food products and 37 °C for therapy.

The obtained OD data were analyzed using a rapid and sensitive data analytic tool, PHIDA. PHIDA was designed and implemented in this study to improve the efficiency and the accuracy of detecting and comparing the host range patterns of phages infecting a wide range of bacterial strains. The tool consists of two spread sheets: the first builds a regression model per each replicate to identify the OD growth trend. This sheet is organized in a dynamic architecture that allows flexibility in adding more strains, phages, or extending the experiment time. Lookup functions combined with conditional functions were used to automatically detect the host range patterns. The second sheet tracks and uses the information, patterns, and values derived by the configurations and the formulas of the first sheet to aggregate and organize the values into a final report. Therefore, the host range determination approach used in this study could be applied as a rapid screening technique for discrimination of unique and re-isolated phages. This method could be a useful non-subjective tool to identify broad or narrow host range phages for different phage applications.

Lastly, the host range index (*HR_i_*) was calculated for all phages, which can be defined as a metric for quantitative analysis of the phage host range. Metric values have been useful to quantitatively describe other phage traits, e.g., the virulence index (*V_P_*) that quantifies the phage killing efficacy in liquid culture [30]. Compared to the *V_P_*, the *HR_i_* aims to compare the growth inhibition effect of multiple phages against a defined panel of strains. More recently, Moller, et al. [46] developed a high throughput and quantitative version of the spot test using a 96-well plate format and turbidimetric measurements. Using this approach, the authors designated the strains as sensitive, semi-sensitive, and resistant to the phage infection, and designated a numerical value to the host range based on the OD measurements of the turbidity in the semisolid agar medium. Although high throughput, this approach exhibits the same pitfalls as the classical spot assay, such as: not reflecting the phage–host interaction in liquid culture, overestimation of the host range, and not monitoring the bacterial growth over the time.

Phage isolation from environmental samples traditionally relies on the enrichment of phage particles by introducing the target strain(s). Other methods apply a sequential enrichment using multiple strains to increase the likelihood of isolating broad host range phages [23]. However, no relationship between the enrichment method and the host range breadth has been reported. Using these enrichment methods, a total of 9 *Listeria*, 14 *Salmonella*, 15 environmental *Pseudomonas*, and 5 clinical *Pseudomonas* infecting phages were isolated, and their host range was quantified and compared using PHIDA. High variability in *HR_i_* values was observed among phages isolated with different enrichment methods against all targeted bacteria. Because a significant proportion of published *Listeria* phages to date are temperate and have narrow host ranges [47], approaches that are biased towards isolation of lytic broad host range phages are desirable. One such approach is the sequential enrichment of environmental samples with strains belonging to distinct serotypes [23]. *Listeria* phages isolated in this study using the CS enrichment method completely inhibited the growth of most strains against which they were screened for the 40 h duration of the experiment (Figure 4, Table S3). On the other hand, SS enrichment yielded two *Listeria* phages: one with a broad and one with a narrow spectrum of activity. CKA13, isolated by SS enrichment, delayed the growth of the 1/2a serotype *Lm*11 but had no effect on the 4b serotype *Lm37*. In contrast, all of the broadly active *Listeria* phages tested completely inhibited the growth of these two strains belonging to distinct lineages [48].

Notably, the newly isolated broad host range *Listeria* phages also inhibited the growth of several non-pathogenic *Listeria* species, including *L. innocua*, *L. seeligeri*, *L. welshemeri*, and *L. grayi* (Table S3), which is consistent with the productive infection of these strains by the phages. Consistent with a previous report that Listex P100 does not infect *L. grayi* [47], the growth of *Lg*127 was not inhibited by this commercially available phage.

The four phages listed in Figure 4 that were isolated by the CE method are all broad host range. However, phage isolation by this technique is relatively labor intensive, because phage lysate has to be plated on lawns of all the strains used in the enrichment. This can result in frequent re-isolation of the same phage since a given phage can produce distinct plaque morphologies on different hosts. Moreover, narrow host range phages might also be isolated by cocktail enrichment [49], which is not optimal for biocontrol applications.

Broad host range *Listeria* phages, such as the ones isolated in this study, belong to the genus *Pecentumvirus*. In the case of phage A511, another member of this genus, the primary receptor for phage attachment was initially reported to be the peptidoglycan (PG) itself, specifically the *N*-acetylglucosamine in the PG [50]. However, a subsequent study identified rhamnose and *N*-acetylglucosamine in the WTA of 1/2a strains of *L. monocytogens* as essential to the infection and binding of A511 receptor-binding proteins to the host. We expect a similar aspect to be true for our broad host range *Listeria* phages, since they can inhibit growth of both 1/2a and 4b strains and since the latter characteristic is a particularity of members of the genus *Pecentumvirus* [51]. Resistance in *L. monocytogenes* to broad host range phages has been reported to be associated with loss of glycosylation of wall teichoic acids (WTAs), which also serve as receptors for phage attachment by serotype-specific phages [50]. In the case of a strain belonging to serovar 4b, the loss of galactose was observed in resistant mutants that were isolated against the pecentumvirus A511 [52]. For serotype 1/2 strains, such as the 1/2a strain *L. monocytogenes* ATCC19111 that was used as the propagation host for the broad host range *Listeria* phages used in this study, the loss of *N*-acetyl glucosamine and rhamnose in the WTA of mutants resistant to broad host range *Listeria* phages was observed [31].

A notable finding from our work is that none of the broadly lytic *Listeria* phages had any effect on the growth of *Lm38* under the conditions tested (Table S3). This strain has previously been serotyped as 1/2b, the strains of which have identical somatic antigens as the serotype 1/2a strains of *Listeria*. The observation that a 1/2 serotype strain is resistant to infection by non-serotype-specific phages is consistent with a mechanism of resistance that comes after receptor binding. One possibility is that this strain contains a functional CRISPR-Cas system. Sequences typical of CRISPR systems have been identified in *L. monocytogenes* in a screen of 128 strains encompassing diverse serotypes [53].

Similarly, *Salmonella* phages isolated with the CS method showed an overall broader host range compared to those isolated with CE and SS. Interestingly, the CS method also led to the isolation of both narrow (SP11) and broad (SP8) host range phages. The presence of abortive infection and exclusion mechanisms in the hosts used for the enrichment step could have led to the isolation of narrow host range phages with the CS method, since phage particles could still be present and carried throughout the enrichment series [1,54]. In contrast, since *Salmonella* serovars belonging to different serogroups (O:9, O:4, O7, and O:8) were used for the CS and CE method and they possess different somatic antigen (O antigen) structures [55,56], it is possible that broad host range phages isolated with this enrichment method might use a conserved surface glycan as a primary receptor, thus allowing the infection of multiple serovars [57]. Broad host range *Salmonella* phages have been reported to use conserved surface structures as receptors, including *N*-acetylglucosamine (GlcNAc) residue of the LPS core [58], core saccharides of the LPS core [59], ferrichrome transporter (FhuA) [60], vitamin B transporter (BtuB) [57], and outer membrane protein C (OmpC) [61]. Conversely, narrow host range phages (e.g., SP11 and SP12) were only able to inhibit the growth of a limited number of serovars, implying the use of more unique surface receptor such as serovar-specific O antigen saccharides or flagellum, which are very diverse among the serovars tested in this study [56,57]. Phages isolated with the CE (SP1–4) method exhibited an almost identical host range; this could be an indication that these phages are re-isolates or closely related phages. In contrast to *Listeria* and EP phages which completely inhibited the bacterial growth of the tested strains (more “C” designations, higher *I_s_* and *HR_i_*), isolated *Salmonella* phages were predominantly able to only delay the bacterial growth of the tested serovars for a period less than the duration of the experiment (24 h). This might be expected since *S. enterica* subsp. *enterica* serovars possess a wide variety of phage resistance mechanisms, including receptor mutation [62], R-M type III systems [63,64], CRISPR-Cas [65], and prophage mediated repression [66].

The EP phages isolated in this study had a high variation in their host range, ranging from an *HR_i_* of 0.0011 to 0.67 (Figure 6, Table S5). In contrast to *Listeria* and *Salmonella* phages, the CE method yielded the phage with the broadest host range (EP1, *HR_i_* 0.67). There was also a high variability when comparing phages isolated against cocktail A (EP1, EP8–10) and cocktail B (EP2–7, and EP11). Cocktail A was comprised of three phylogenetically distant *Pseudomonas* species [67]: *P. fluorescens, P. putida,* and *P. mendocina* [68]. Overall, cocktail A led to the isolation of phages with a narrower host range compared to cocktail B. Species diversity in cocktail A maybe have contributed to the isolation of both broad (EP1) and narrow (EP8–10) phages. EP1 showed growth inhibition against all *Pseudomonas* species included in cocktail A, whereas EP8–10 only exhibited growth inhibition against *P. aeruginosa* species. On the other hand, cocktail B was comprised of *P. aeruginosa* environmental isolates and yielded phages with higher *HR_i_* compared to cocktail A. As expected, phages isolated with cocktail B inhibited the growth of mainly *P aeruginosa* species and showed a broader host range, since the *Pseudomonas* panel used in this study mainly comprised the *P. aeruginosa* species (17/22). Interestingly, delay in the bacterial growth was not observed in any of the strains infected with EP11, which was isolated with the CE method. A slight decrease in the OD_max_ of ~20% was observed in *P. aeruginosa* ATCC33348 when infected with EP11. This behavior is commonly associated with filamentous phages (Pf phages) such as *P. aeruginosa* phage Pf4 [69]. Pf phages are known to cause chronic infection in which viral progeny is released from the infected cells without lysis; this can be reflected as a slight growth inhibition (in this case, reduced OD_max_) [70,71]. On the other hand, phages isolated with the CS method (EP12–15) showed comparable *HR_i_* with the EP14 phage exhibiting the highest *HR_i_* of 0.38. These phages were isolated using a series of isolates of *P. aeruginosa*, thus phage isolation was biased towards the enrichment of phages inhibiting the growth of this species.

Conversely, only five CP phages were isolated using both the CE and CS methods. Among these phages, CP1 showed the highest *HR_i_* of 0.37 and was isolated using the CE method. Similarly, the CE method led to the isolation of narrow (CP4) and broad (CP1) phages. Except CP4, all phages (CP1–CP3) were able to inhibit the growth of all the LES-like isolates present in the enrichment cocktail. LES isolates possess highly similar core genomes with a relatively low number of single nucleotide polymorphisms (SNPs), accessory elements, and prophages [36]. Therefore, phages capable of infecting these isolates might rely on core genes to complete the infection cycle and might be able to overcome prophage mediated repression and exclusion [36,72,73]. Additionally, these phages may utilize conserved structures as receptor such as Type IV pili (present in all *P aeruginosa*) or saccharides present in the core LPS [74,75]. Similarly, as EP11, CP4 showed a weak growth inhibition against LES-like isolates, suggesting that this phage may cause chronic infection [70,71]. Interestingly, the CS method led to the isolation of one narrow host range phage (CP5). CP5 exhibited growth inhibition against four of the tested strains including only two of the four strains used in the enrichment series.

Our results suggest that broad host range phages isolated in this study using the three enrichment methods may possess specialized receptor binding proteins (RBPs) that recognize one or more highly conserved receptors allowing them to infect multiple strains [76]. Additionally, these phages might have evolved to completely or partially overcome phage resistance mechanisms, including restriction–modification (R–M) and CRISPR-Cas systems, receptor masking, and repression by prophages [66,73,77,78].

## 5. Conclusions

In conclusion, the study did not find a direct correlation between the enrichment method used for phage isolation and the host range spectrum of the isolated phages in any of the three bacteria genera. Except for *Listeria*, all methods yielded phages with narrow and broad host range. In short, one enrichment approach cannot fit all target bacteria and cannot be recommended for isolating broad or narrow host range phages. Considerations such as labor intensity and time consumption must be taken when considering these enrichment methods for phage isolation. For example, single strain enrichment can be adequate when time and resources are a constraint, whereas including all SS, CS, and CE in the isolation pipeline can be used to maximize the likelihood of isolating broad host range phages. The high throughput method and the analytics tool developed in this study can be implemented to quantitatively determine the host range of any phage and provide a standard for selecting phages for different applications. Additionally, these tools can be used in the future to develop more sophisticated analysis software integrating artificial intelligence to predict host range profile and phage-host interactions.

## Data Availability

Not applicable.

## Supplementary Materials

The following are available online at https://www.mdpi.com/article/10.3390/v13112120/s1, Figure S1: Plaque assay results of phage isolates against *Listeria* spp., Table S1. List of bacterial strains used in this study, Table S2: Enrichment method used for isolation of phages in this study. Table S3: Host range determination of *Listeria* phages isolated using different enrichment methods. Table S4: Host range determination of *Salmonella* phages isolated using different enrichment methods. Table S5: Host range determination of *Pseudomonas* spp. Environmental phages isolated using different enrichment methods. Table S6: Host range determination of *Pseudomonas* spp. Clinical phages isolated using different enrichment methods.
