# Supplementary material for: PHIDA: A High Throughput Turbidimetric Data Analytic Tool to Compare Host Range Profiles of Bacteriophages Isolated Using Different Enrichment Methods
Source: Viruses. 2021 Oct 21;13(11):2120. doi: 10.3390/v13112120 (PMC8623551; doi:10.3390/v13112120)
Supplement: Supplementary file 1 [file viruses-13-02120-s001.zip › viruses-1407185-supplementary.pdf]

viruses

MDPI

Table S1. List of bacterial strains used in this study.

Bacterial Strains	Strain ID number	Source of Strains
Listeria **spp.**		
		GRDC
		GRDC
L. monocytogenes	HA2018008	GRDC
L. monocytogenes	HA2018009	ATCC
L. monocytogenes	HA2018010	GRDC
L. monocytogenes 1/2a	ATCC1911	UoG
L. monocytogenes	HA2018012	UoG
L. monocytogenes 4b	HA2018037	UoG
L. monocytogenes 1/2b	HA2018038	UoG
L. monocytogenes	HA2018039	UoG
L. monocytogenes	HA2018040	UoG
L. monocytogenes 1/2a	HA2018041	GRDC
L. monocytogenes	HA2018042	GRDC
L. monocytogenes 4b	HA2018072	UoG
L. monocytogenes 4b	HA2018073	UoG
L. monocytogenes	HA2018111	UoG
L. monocytogenes	HA2018112	GRDC
L. monocytogenes	HA2018113	GRDC
L. monocytogenes 4b	HPD 4493	UoG
L. monocytogenes 4b	ATCC19115	UoG
L. innocua	HA2018115	UoG
L. innocua	HA2018116	UoG
L. innocua	HA2018117	UoG
L. seeligeri	HA2018119	UoG
L. seeligeri	HA2018121	UoG
L. welshimeri	HA2018123	UoG
L. grayi	HA2018127	UoG
L. ivanovii	HA2018130	UoG
Salmonella enterica subsp. **Enterica**		
S. Enteritidis	ATCC13076	ATCC
S. San Diego	SA20143137	PHAC-NML
S. Heidelberg	SA20024302	GRDC
S. Schwarzengrund	HA2018050	UoG
S. Panama	SA20110471	PHAC-NML
S. Typhimurium	ATCC14028	ATCC
S. Stanley	SA20112218	PHAC-NML
S. Saintpaul	HA2018035	UoG
S. Typhimurium (DT104)	HA2018005	GRDC
S. Infantis	HA2018049	UoG
S. Javiana	SA20161974	PHAC-NML
S. Thompson	ATCC8391	ATCC
S. Anatum	HA2018046	UoG
S. Senftenberg	HA2018045	UoG
S. Hadar	HA2018051	UoG
S. Orainenberg	HA2018036	UoG
S. Newport	ATCC6962	ATCC
S. Montevideo	HA2018047	UoG
S. Mbandaka	HA2018048	UoG
S. Reading	HA2018044	UoG
S. Derby	HA2018043	UoG
Pseudomonas **spp.**		
P. aeruginosa	ATCC33348	ATCC

P. aeruginosa	ATCC33349	ATCC
P. aeruginosa	ATCC33350	ATCC
P. aeruginosa	ATCC33351	ATCC
P. aeruginosa	PAO1	UoG
P. aeruginosa	ATCC43731	ATCC
P. aeruginosa	ATCC43732	ATCC
P. aeruginosa	SG81	UoG
P. aeruginosa	SG81R	UoG
P. aeruginosa	PA14	UoG
P. aeruginosa	7700	UoG
P. aeruginosa	228	UoG
P. protegens	Pf5	AAFC
P. fluorescens	Pf4A3	AAFC
P. fluorescens	A506	AAFC
P. putida	GTA-PS01	CFIA
P. aeruginosa	GTA-PS02	CFIA
P. aeruginosa	GTA-PS03	CFIA
P. aeruginosa	GTA-PS04	CFIA
P. aeruginosa	GTA-PS05	CFIA
P. mendocina	GTA-PS10	CFIA
P. aeruginosa	LES-like 1	UoG
P. aeruginosa	LES-like 4	UoG
P. aeruginosa	LES-like 5	UoG
P. aeruginosa	LES-like 7	UoG
P. aeruginosa	LES-like B58	UoG
P. aeruginosa	LES-like B65	UoG
P. aeruginosa	LES-like 430	UoG
P. aeruginosa	LES-like 431	UoG
P. aeruginosa	GTA-PS02	CFIA
P. aeruginosa	GTA-PS03	CFIA
P. aeruginosa	GTA-PS04	CFIA
P. aeruginosa	SG81	UoG
P. aeruginosa	SG81R	UoG
P. putida	A460	CFIA
P. aeruginosa	PA14	UoG

Abbreviation: ATCC, American Type Culture Collection; PHAC-NML, Public Health Agency of Canada National Microbiology Laboratory, Guelph; UoG, University of Guelph, American Type Culture Collection; CFIA.

Table S2. Enrichment method used for isolation of phages in this study.

Strain	Enrichment	Phage	Enrichment details	Strain	Enrichment	Phage	Enrichment details
Listeria spp	**CE**			***Pseudomonas* spp. Environmental**	**CE**		**Cocktail A:** *P. fluorescens* GTA-PS05, *P. putida* A460, *P. mendocina* GTA-PS10, *P. fluorescens* 4A3, *P. fluorescens* A506 **Cocktail B:** *P. aeruginosa*: 770 (**A**), 228 (**B**), GTA-PS02, GTA-PS03, GTA-PS04
	Cocktail	CKA15	*Lm*4b, *Lm*1/2b, *L. innocua*		Coctail A	EP1	
	Cocktail	CKA16	*Lm*4b, *Lm*1/2b, *L. innocua*		Cocktail B	EP2	
	Cocktail	CKA17	*Lm*4b, *Lm*1/2b, *L. innocua*		Cocktail B	EP3	
	Cocktail	CKA18	*Lm*4b, *Lm*1/2b, *L. innocua*		Cocktail B	EP4	
	**CS**				Cocktail B	EP5	
		CKA4	*Lm11*(1/2a)→*Lm 37* (4b)→*Lin115*→*Lm11* (1/2a)		Cockail B	EP6	
		CKA8	Same as CKA4		Cocktail B	EP7	
		CKA11	Same as CKA4		Cocktail A	EP8	

	**SS**				Cocktail A	EP9	
		CKA13	*Lm11* (1/2a)		Cocktail A	EP10	
		CKA14	*Lm11* (1/2a)		Cocktail B	EP11	
***Salmonella* spp.**	**CE**		**Cocktail**	***Pseudomonas* spp. Clinical**	**CS**		**Series A:** A→B→*P.a* PA01→*P.a* ATCC3348→*P.a* ATCC43731
	Cocktail	SP1	S. Enteritidis (**A**), S. Newport (**B**), S. Infantis (**C**), S. Heidelberg (**D**), S Typhimurium (DT104) (**E**)		Series A	EP12	
	Cocktail	SP2			Series A	EP13	
	Cocktail	SP3			Series A	EP14	
	Cocktail	SP4			Series A	EP15	
	CS		**Series 1:** A→B→C→D→E **Series 2:** E→A→B→C→D→E **Series 3:** C→E→B→D→C **Series 4:** D→C→E→B→E **Series 5:** B→D→C→E→A→B		**CE**		**Cocktail A:** *P.a* LES isolates: 1, 4, B58, 3B2
	Series 1	SP5			Cocktail A	CPP1	
	Series 1	SP6			Cocktail A	CPP2	
	Series 2	SP7			Cocktail A	CPP3	
	Series 3	SP8			Cocktail A	CPP4	
	Series 4	SP9			**CS**		**Series A:** LES isolates: 5→7→58→1
	Series 5	SP10			Series A	CPP 5	
	Series 2	SP11					
	**SS**						
	S. Enteritidis	SP12					
	S. Infantis	SP13					
	S. Enteritidis	SP14					

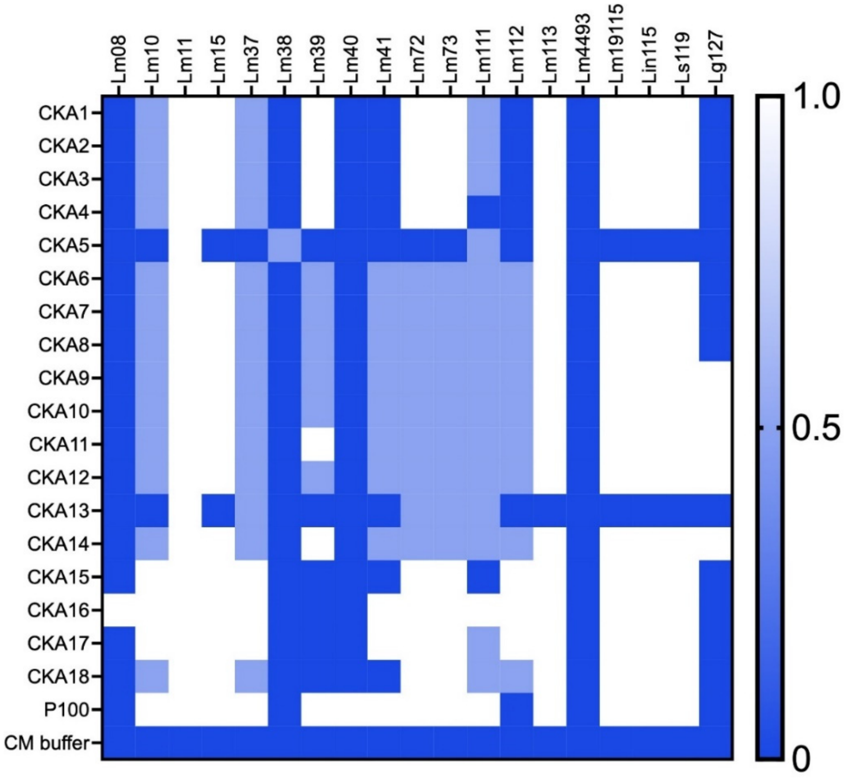

Figure S1. Plaque assay results of phage isolates against *Listeria* spp. Gradient indicates degree of lytic activity. Value of 1 corresponds to strong lytic activity, 0.5 weak activity, 0 no activity. Seven microliters phage suspension at 10^4PFU/ml on *L. monocytogenes* ATCC19111 were spotted on lawns of the various strains.

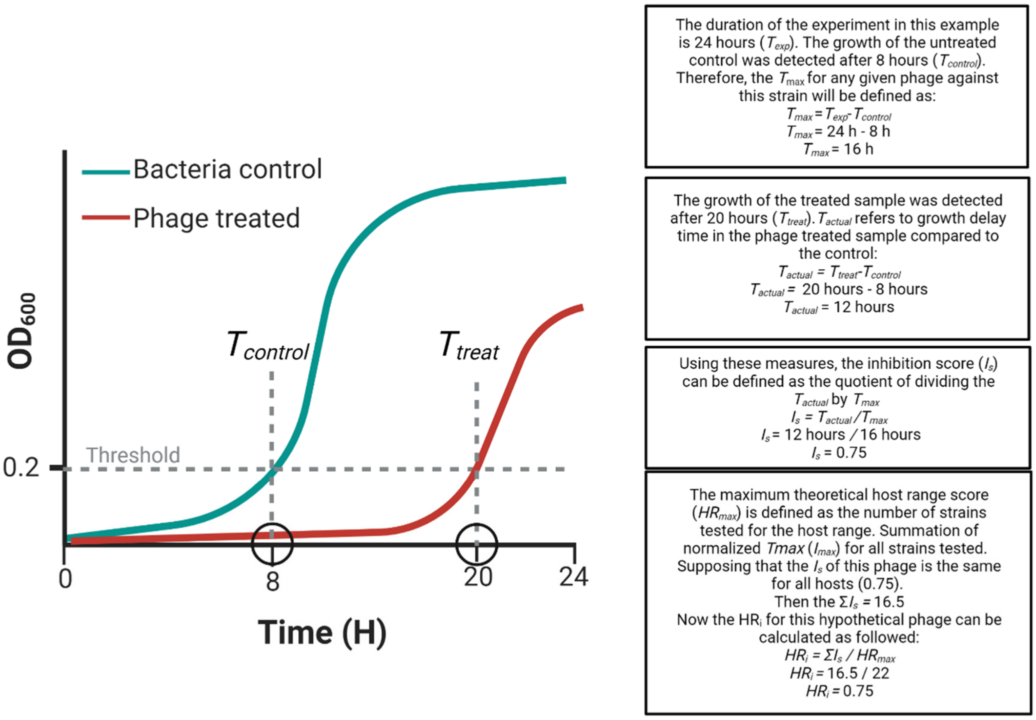

Figure S2. Example of the calculation of the Host Range Index (HR_i).

Table S3. Host range determination of *Listeria* phages isolated using different enrichment methods.

**Bacterial strains**	**Cocktail enrichment (CE)**				**Cyclic sequential enrichment (CS)**			**Single serovar enrichment (SS)**		**Reference phage**
	**CKA15**	**CKA16**	**CKA17**	**CKA18**	**CKA4**	**CKA8**	**CKA11**	**CKA13**	**CKA14**	**P100**
*Lm*08	N	D+	N/L+	N	N	N	N	N	N	N
*Lm*09	C	C	C	C	C	C	C	D+	C	C
*Lm*10	C	C	C	D+	C	C	C	N	C	C
*Lm*11	C	C	C	C	C	C	C	D+	C	C
*Lm*37	C	C	C	C	C	C	C	N	C	C
*Lm*38	N	N	N	N	N	N	N	N	N	N
*Lm*39	N	N	N	N	C	D+	D+	N	C	C
*Lm*41	N	N	N	N	D+	C	C	D	D+	C
*Lm*72	D+	D+	D+	D+	C	C	C	N	C	C
*Lm*73	C	C	C	D+	C	D+	C	N/L	C	C
*Lm*111	D+	C	C	C	C	C	C	N	C	C
*Lm*112	D+	D+	D+	D+	N	C	C	N	C	C
*Lm*113	D+	D+	C	N	C	C	D+	D	C	N
*Lm*19115	C	C	C	C	C	C	C	N	C	C
*Ls*119	D+	C	C	C	C	C	C	N	C	C
*Ls*121	N	N	N	N	C	C	C	N	C	C
*Lw*123	D+	C	C	D+	C	C	C	D	C	C
*Lg*127	C	C	D+	C	N	D	N	N/L	N	N
*Lin*115	C	C	C	C	C	D+	C	N	C	C
*Lin*116	C	C	C	C	D	D	C	D	C	C
*Lin*117	N/L+	N/L+	N/L+	N	N/L	N/L+	C	N/L	C	N
*Liv*130	N/L+	N/L	N/L+	N	N/L	N/L	C	N	C	N

HRHR_i	0.63	0.70	0.66	0.59	0.68	0.72	0.84	0.80	0.86	0.75

Designation: C, complete inhibition, D+, >5 h delay to reach exponential phase; D, <5 h delay to reach exponential phase; N, no effect; HR_i, host range index. Abbreviation: *Lm, Listeria monocytogenes; Ls, Listeria seeligeri; Lw, Listeria welshimeri; Lg, Listeria grayi; Lin, Listeria Innocua,.*

Table S4. Host range determination of *Salmonella* phages isolated using different enrichment methods.

**Bacterial strains**	**Cocktail enrichment (CE)**				**Cyclic sequential enrichment (CS)**							**Single serovar enrichment (SS)**		
	**SP1**	**SP2**	**SP3**	**SP4**	**SP5**	**SP6**	**SP7**	**SP8**	**SP9**	**SP10**	**SP11**	**SP12**	**SP13**	**SP14**
S. Enteritidis	D+	D	D	D	D+	D	D+	N	N	N	N	D+	N	D+
S.Typhimurium	D	D	D	D	D+	D	D+	D	D+	N	D	D	N	D
S. Heidelberg	D	D	D	D	D	D+	D	D+	D+	D+	D+	D	N	D
S. Infantis	N	N	N	N	D+	D+	N	C	D+	D+	D	D	C	N
S. Thompson	N	N	N	N	C	C	D	C	D+	N	N	N	C	N
S. Newport	N	N	N	N	D	D	N	D+	N	D	D	N	N	N
S. Schwarzengrund	D+	D+	D+	D+	D+	D+	D+	D	N	N	N	D+	N	D+
S. Stanley	D	D	D	D	D	D	D	D+	D+	D+	D+	N	N	D
S. Saintpaul	D	D	D	D	D	D	D	D+	D	D+	D	N	N	D
S. Typhimurium (DT104)	N	N	N	N	D	D	D	D	D	D+	D	N	D+	N
S. Panama	D+	C	D+	C	D	D	C	N	N	N	N	D+	N	D
S. Javiana	N	N	N	N	D	D	D	D	D	D	N	N	N	N
S. Anatum	D+	D+	D+	D+	N	N	D+	N	D	N	N	N	N	D+
S. Senftenberg	D+	D+	D+	D+	N	N	D+	N	N	N	N	N	N	D+
S. Senftenberg	D+	D+	D+	D+	D	D	D+	N	N	D	N	N	N	D+
S. Hadar	N	N	N	N	D	D	D	D+	D+	D+	D+	N	N	N
S. Orainenberg	N	N	N	N	D+	D+	N	N	N	N	N	N	C	N
S. Montevideo	N	N	N	N	D	D	N	N	N	N	N	N	D	N
S. Mbandaka	N	N	N	N	N	N	N	N	N	N	N	N	N	N
S. Reading	N	N	N	N	N	N	N	N	N	N	N	D	N	N
S. Derby	N	N	N	N	N	N	N	N	N	N	N	N	N	N
S. San Diego	D	D	D	D	D	D	D	D+	D	D	D	D+	N	D
HR_i	0.13	0.13	0.13	0.14	0.24	0.19	0.20	0.21	0.11	0.12	0.07	0.08	0.17	0.11

Designation: C, complete inhibition, D+, >5 h delay to reach exponential phase; D, <5 h delay to reach exponential phase; N, no effect; HR_i, host range index. Abbreviation: *S, Salmonella.*

Table S5. Host range determination of *Pseudomonas spp.* Environmental phages isolated using different enrichment methods.

**Bacterial strains**	**Cocktail enrichment (CE)**											**Cyclic sequential enrichment (CS)**			
	**EP1**	**EP2**	**EP3**	**EP4**	**EP5**	**EP6**	**EP7**	**EP8**	**EP9**	**EP10**	**EP11**	**EP12**	**EP13**	**EP14**	**EP15**
Pa ATCC33348	C	D+	D+	D+	D+	D+	N	D+	D+	N	N/L	D+	D+	D+	D+
Pa ATCC33349	C	D+	D	D+	C	C	C	N	N	N	N	D+	D+	C	D
Pa ATCC33350	C	D+	N	D+	D+	D+	D+	D+	D	D+	N	D+	D+	C	D+
Pa ATCC33351	D+	N	D	N	N	N	N	N	N	N	N	D	N	N	N
Pa PAO1	C	D+	C	D+	D+	D+	C	D+	C	D+	N	D+	N/L	D+	D+
Pa ATCC43731	C	D+	N	C	C	D+	D+	N	N	N	N	D+	D+	C	N
Pa ATCC43732	D	D+	N/L	D+	D+	D+	D+	N	N	N	N	D+	N	D+	N

Pa SG81	D+	D	N	N	N	N	N	N	N	N	N	D	N	N	N
Pa SG81R	D+	D+	N	D	N	D	N	N	N	N	N	D+	D+	N	N/L
Pa PA14	D	N/L	N	N	N	N	D	N	D+	D	N	D	N/L	N	N
Pa 7700	D+	C	D+	D+	D+	D+	N	D+	N	D	N	N	D	C	D+
Pa 228	D+	D+	D+	D+	D+	D+	D+	D+	D	D+	N	D+	D+	D+	D+
Pp Pf5	C	D+	N	N	N	N	N	C	N	N	N	N	D+	N	N
Pf Pf4A3	C	N/L	N	N	N	N	N	N	N	N	N	N	D	N	N
Pf A506	D+	N	N	N	N	N	N	N	N	N	N	N	N	N	N
Pm GTA-PS01	D+	N	D+	N	N	N	N	N	N	N	N	N	N	N	D+
Pa GTA-PS02	D+	D+	N	N	N	N/L	N	D+	D+	D+	N	D+	N	N	N
Pa GTA-PS03	D+	N	D	N	N	N	N	N	N	N	N	D	N	N	N
Pa GTA-PS04	D+	D+	N	N	N	N	N	N	N	N	N	D+	N	N	N
Pa GTA-PS05	D+	N	D+	N	N	N	N	N	N	N	N	N	N	D+	D
Pa GTA-PS10	N	N	N	N	N	N	N	N	N	N	N	N	N	N	N
E. coli K-12	D+	N	N	N	N	N	N	N	N	N	N	N/L	N	N	N
HR_i	0.67	0.41	0.28	0.31	0.32	0.32	0.24	0.26	0.16	0.17	0.00	0.25	0.28	0.38	0.27

Designation: C, complete inhibition, D+, >5 h delay to reach exponential phase; D, <5 h delay to reach exponential phase; N, no effect; HR_i, host range index. Abbreviation: Pa, *Pseudomonas aeruginosa,* Pp, *Pseudomonas protegens,* Pf, *Pseudomonas fluorescens,* Pm, *Psedumonas mendocina.*

Table S6. Host range determination of *Pseudomonas spp.* Clinical phages isolated using different enrichment methods.

**Bacterial strains**	**Cocktail enrichment (CE)**				**Cyclic sequential enrichment (CS)**
	**CP1**	**CP2**	**CP3**	**CP4**	**CP5**
Pa LES-like 1	D+	D	C	N	D+
Pa LES-like 4	C	C	C	N	N
Pa LES-like 5	D+	D+	D+	N	C
Pa LES-like 7	D	D	C	D	D+
PaLES-like B58	C	D+	D	N/L	N
Pa LES-like B65	C	D+	D	N/L	N
Pa LES-like 430	C	D	C	N	D
Pa LES-like 431	C	D+	C	N/L	N
Pa 7700	D+	N	N	N	N
Pa 228	D	N	N	N	N
Pa GTA-PS02	D	N	N	N	N
Pa GTA-PS03	D	N	N	N	N
Pa GTA-PS04	N	N	N	N	N
Pa SG81	D+	D+	D	N	D
Pa SG81R	D	N	N	N	N
Pa PA14	D	N	N	N	N
Pf A506	N	N	N	N	N
Pf Pf4A3	N	N	N	N	N
Pa ATCC33348	D	N	N	N	N
Pa ATCC33349	N	N	N	N	N
Pa PAO1	D+	D+	N	N	N
E. coli K-12	N	N	N	N	N
HR_i	0.37	0.21	0.27	0	0.06

Designation: C, complete inhibition, D+, >5 h delay to reach exponential phase; D, <5 h delay to reach exponential phase; N, no effect; HR_i, host range index. Abbreviation: Pa, *Pseudomonas aeruginosa,* Pf, *Pseudomonas fluorescens.*
